# Genome Sequences of Two Naphthalene-Degrading Strains of *Pseudomonas balearica*, Isolated from Polluted Marine Sediment and from an Oil Refinery Site

**DOI:** 10.1128/genomeA.00116-17

**Published:** 2017-04-06

**Authors:** Francisco Salvà-Serra, Hedvig E. Jakobsson, Antonio Busquets, Margarita Gomila, Daniel Jaén-Luchoro, Carolina Seguí, Francisco Aliaga-Lozano, Elena García-Valdés, Jorge Lalucat, Edward R. B. Moore, Antoni Bennasar-Figueras

**Affiliations:** a Department of Infectious Diseases, Sahlgrenska Academy, University of Gothenburg, Gothenburg, Sweden; b Centre for Antibiotic Resistance Research (CARe) at University of Gothenburg, Gothenburg, Sweden; c Microbiology, Department of Biology, University of the Balearic Islands, Palma de Mallorca, Spain; d Microbiology, Clínica Rotger, Palma de Mallorca, Spain; e Culture Collection University of Gothenburg (CCUG), Sahlgrenska Academy, University of Gothenburg, Gothenburg, Sweden

## Abstract

The genome sequences of *Pseudomonas balearica* strains LS401 (CCUG 66666) and st101 (CCUG 66667) have been determined. The strains were isolated as naphthalene degraders from polluted marine sediment and from a sample from an oil refinery site, respectively. These genomes provide essential data about the biodegradation capabilities and the ecological implications of *P. balearica*.

## GENOME ANNOUNCEMENT

*Pseudomonas balearica* was described as a novel species in 1996 (1), and the complete genome sequence of the type strain was recently published (2). Here, we report the draft genome sequences of *P. balearica* strain LS401 (CCUG 66666), isolated as a naphthalene degrader from a polluted marine sediment (Barcelona, Spain) (3), and *P. balearica* strain st101 (CCUG 66667), isolated as a phenanthrene and naphthalene degrader from *Spartina patens* rhizosphere at an oil refinery site (NY/NJ harbor estuary, USA) (4).

Both strains were cultivated at 30°C on Columbia agar base plus 5% horse blood. Genomic DNA was isolated using a Wizard SV genomic DNA purification system (Promega, Madison, WI, USA). DNA was sequenced with an Illumina HiSeq 2500 instrument, generating paired-end reads of 126 bp and an insert size ranging from 130 to 680 bp. Reads were trimmed with Sickle version 1.33 (5) and error-corrected with SPAdes version 2.4.0 (6). A first *de novo* assembly was obtained using 6,825,396 reads of each strain and Velvet version 1.2.10 (7). The genome sequence of *P. balearica* DSM 6083^T^ was used as a reference to obtain a second assembly for both strains by mapping with CLC Genomics Workbench version 8 (CLC bio, Aarhus, Denmark). A consensus genome assembly of each strain was obtained by combining the *de novo* and the reference-based assemblies with Metassembler version 1.5 (8). Assemblies were assessed using QUAST version 3.1 (9) and Feature Response Curves (10). The genome sequence of *P. balearica* LS401 is formed by 60 contigs with a total length of 4,293,681 bp. The largest contig is 358,463 bp, and the G+C content is 65.0%. The genome sequence of *P. balearica* st101 consists of 45 contigs with a total length of 4,363,152 bp. The largest contig is 805,012 bp and the G+C content is 64.9%, which is similar to the 64.7% of the type strain (2). Analyses by average nucleotide identity based on BLAST (ANIb) (11), using JSpecies version 1.2.1 (12), among the genome sequences of *P. balearica* strains LS401, st101, and DSM 6083^T^ ranged from 97.9% to 98.5%.

Both genome sequences were annotated, using the NCBI Prokaryotic Genome Annotation Pipeline (PGAP) (13). Annotation of *P. balearica* LS401 identified 4,087 genes, including 4,026 coding sequences (CDSs) and 54 tRNA genes. Annotation of *P. balearica* st101 revealed 4,093 predicted genes, including 4,033 CDSs and 53 tRNA genes. Highly conserved genes for chemotaxis and flagellum synthesis were identified. The complete naphthalene degradation upper and lower pathways (14, 15) were found in the genome of *P. balearica* LS401 but not in *P. balearica* st101. The capability of *P. balearica* st101 to degrade naphthalene and phenanthrene could be explained by the presence of a complete homogentisate degradation pathway, which is present in both genomes. Genes for complete denitrification and for the benzoate degradation pathway were found in both genomes. A CRISPR-Cas system type I, subtype I-E (16), was found in the genome of *P. balearica* st101 using CRISPRFinder (17).

### Accession number(s).

These whole-genome shotgun projects have been deposited in DDBJ/ENA/GenBank under the accession numbers LONE00000000 and LONF00000000. The versions described in this paper are the second versions, LONE02000000 and LONF02000000.
